# Thermally stimulated exciton emission in Si nanocrystals

**DOI:** 10.1038/lsa.2017.133

**Published:** 2018-01-26

**Authors:** Elinore MLD de Jong, Huub Rutjes, Jan Valenta, M Tuan Trinh, Alexander N Poddubny, Irina N Yassievich, Antonio Capretti, Tom Gregorkiewicz

**Affiliations:** 1 Van der Waals-Zeeman Institute, University of Amsterdam, Science Park 904, 1098 XH, Amsterdam, The Netherlands; 2 Department of Chemical Physics and Optics, Faculty of Mathematics and Physics, Charles University, Ke Karlovu 3, 121 16 Prague 2, Czech Republic; 3 Department of Electrical Engineering and Computer Science, University of Michigan, 2200 Bonisteel Blvd, Ann Arbor, MI 48109, USA; 4 Ioffe Institute, Russian Academy of Sciences, 26 Polytechnicheskaya, 194021 St Petersburg, Russia

**Keywords:** micro-photoluminescence, nanocrystal, optical spectroscopy, silicon, thermal

## Abstract

Increasing temperature is known to quench the excitonic emission of bulk silicon, which is due to thermally induced dissociation of excitons. Here, we demonstrate that the effect of temperature on the excitonic emission is reversed for quantum-confined silicon nanocrystals. Using laser-induced heating of silicon nanocrystals embedded in SiO_2_, we achieved a more than threefold (>300%) increase in the radiative (photon) emission rate. We theoretically modeled the observed enhancement in terms of the thermally stimulated effect, taking into account the massive phonon production under intense illumination. These results elucidate one more important advantage of silicon nanostructures, illustrating that their optical properties can be influenced by temperature. They also provide an important insight into the mechanisms of energy conversion and dissipation in ensembles of silicon nanocrystals in solid matrices. In practice, the radiative rate enhancement under strong continuous wave optical pumping is relevant for the possible application of silicon nanocrystals for spectral conversion layers in concentrator photovoltaics.

## Introduction

Silicon (Si) is currently the most important semiconductor material for electronic and photovoltaic applications. However, its light absorption and emission rates are lower than those of direct bandgap materials. The optical properties of Si can be improved using nanostructures, where quantum confinement effects play a role, resulting in the opening up of the indirect bandgap and an increase in the radiative recombination rate, among other effects^1, 2, 3, 4, 5^. Simultaneously, laser heating during optical excitation is strongly enhanced in nanostructures compared to the bulk^6^. The heating of nanocrystals (NCs) occurs due to phonon production, which can originate from, among others, (i) cooling of hot carriers generated by over-bandgap excitation, (ii) emission by indirect bandgap recombination to compensate for the momentum mismatch, and/or (iii) non-radiative Auger recombination of multiple electron-hole pairs appearing in a single Si NC^7, 8^. The latter process sets the upper emissivity limit of a single photon per NC. In this study, we investigated the effect of heating on the excitonic emission of Si NCs embedded in a SiO_2_ matrix, and we demonstrated an effective threefold enhancement in their radiative recombination rate. This result represents a proof-of-principle demonstration that purposeful phonon management can be used to manipulate the optical properties of Si NCs to increase the optical faculty of Si.

## Materials and methods

### Materials

In this study, samples of closely packed Si NCs embedded in a SiO_2_ matrix were fabricated using two different preparation methods, namely, radio-frequency co-sputtering and plasma-enhanced chemical vapor deposition (PECVD). The samples were prepared by radio-frequency co-sputtering of Si and SiO_2_ on a quartz substrate, followed by thermal annealing in nitrogen (N_2_) gas at 1150 °C. The samples exhibited a multilayer (ML) structure with a total of 100 bilayers, each consisting of a 5-nm passive SiO_2_ and a 3.5-nm active layer containing the Si NCs (see Ref. 9 for further details). For comparison, and to create a general picture that is not linked to a particular fabrication method, a set of samples grown by PECVD was also investigated. These samples were deposited as alternating layers of Si-rich silicon oxynitride (SRON; SiO_x_N_y_) and stoichiometric SiO_2_ on fused silica substrates, followed by annealing in high-purity N_2_ gas at 1150 °C and in hydrogen (H_2_) gas at 500 °C. The PECVD ML samples all had stoichiometry parameters (x and y), which were nearly identical and each contain 40 bilayers of 4.5-nm SRON and an SiO_2_ spacer thicknesses of 1, 1.6, 2.2 or 2.8 nm for samples ML1 to ML4, respectively (see Refs. 10, 11 for further details).

### Photoluminescence

The time-integrated PL spectra of the co-sputtered samples were obtained under 405 nm continuous wave (cw) excitation (Mitsubishi ML320G2-11) in an inverted microscope setup (Zeiss Axio Observer Z1) with an objective (Zeiss LD EC Epiplan-Neofluar 100 × /0.75 DIC). Part of the sample was selected using a slit, and the PL was detected using a liquid N_2_-cooled charge-coupled device (CCD, Princeton Instruments PyLoN: 1340 × 400B) coupled to a spectrometer (Princeton Instrument Acton SP2300). Replacing the slit and the grating with a mirror allowed us to resolve the beam shape and the intensity profile of the beam incident on the sample. The laser power was adjusted using a set of neutral-density filters and a Glan-Thompson polarizer.

The power dependence of the PL of the PECVD samples was studied using a home-built microspectroscope based on an inverted optical microscope (Olympus IX-71) with an objective lens (100 × /0.8). Excitation was provided by either a 405 nm cw diode laser (Omicron LDM405.120.CWA.L) or the 355 nm output of a frequency-tripled diode-pumped Nd:YAG laser (CNI Optoelectronics MRL-F-355, repetition rate of 4 kHz and pulse width of approximately 6 ns) coupled to the objective in the epifluorescence configuration. The PL from the excitation spot was imaged on the entrance slit of a 30-cm imaging spectrometer (Acton SpectraPro SP-2358i) with a back-illuminated liquid N_2_-cooled CCD (Princeton Instruments SPEC-10:400B) as the detector.

An important advantage of these two imaging spectroscopy arrangements is that the PL detection area can be precisely controlled, avoiding averaging effects that occur when the whole excitation spot is detected (as in a standard PL setup). Moreover, the spot size is kept constant for the different excitation powers; this is significant since the (possible) temperature rise produced by the laser illumination is dependent on the spot size^12^. All measurements were performed in ambient air at room temperature, and all PL spectra were corrected for the sensitivity of the detection system.

### Transient-induced absorption

The transient-induced absorption (IA) experiments were performed with an ultrafast pump-probe setup composed of a Ti:sapphire regenerative amplifier (pulse width of ~100 fs) at a repetition rate of 1 kHz (Spectra-Physics). The fundamental output beam at 800 nm was split into two paths. The first path was directed into an optical parametric amplifier (TOPAS, LightConversion) to generate a tunable pump beam ranging from the ultraviolet to the near-infrared, whereas the second path was focused onto a sapphire crystal to produce the broadband supercontinuum (850–1600 nm, 0.78–1.46 eV) as a probe. The transmitted probe beam was detected by a pair of high-speed multichannel detector arrays coupled to a high-speed data acquisition system (HELIOS, Ultrafast Systems). The IA signal, *I*_IA_, is determined as:


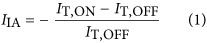


where *I*_T,ON_ (*I*_T,OFF_) is the transmitted probe intensity with the pump laser on (off).

## Results and discussion

### Flux-dependent photoluminescence properties

**Figure 1:**
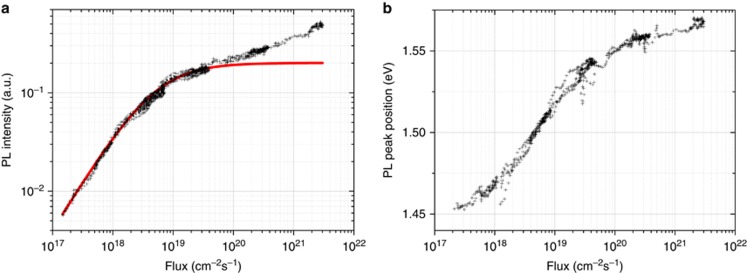
Typical flux dependence of the photoluminescence intensity for the Si nanocrystals in the SiO_2_ sample under 405 nm (3.06 eV) continuous wave excitation. (**a**) The PL intensity (of a co-sputtered sample, *λ*_det_=870 nm, *E*_det_=1.43 eV) at more than four orders of magnitude of the excitation pump flux in double logarithmic representation. The red solid curve corresponds to the behavior expected on the basis of a simple model with an efficient Auger recombination. (**b**) The excitonic PL peak position as a function of the excitation photon flux for the sample depicted in **a**.

In general, the PL intensity in the low-power regime, when none of the NCs have absorbed more than a single photon, is proportional to the number of absorbed photons and is therefore roughly linear to the pump flux (or fluence in the case of pulsed excitation). For a higher flux, the PL growth rate decreases toward zero when multiple excitons appear in a single NC. Calculations for spherical Si NCs show Auger recombination time constants in the range of 0.01 to 1 ns for NCs of similar sizes as investigated in this study (*d*_NC_~2.5–7 nm)^13^. Since the radiative lifetime is much longer (~500 μs), the significantly faster non-radiative Auger recombination process removes all excess carriers so that only a single electron-hole pair per NC will recombine radiatively. Thus, the Auger recombination of multiple excitons sets the upper limit for the number of emitted photons, independent of the excitation photon energy^8^. On the other hand, the number of phonons that can be generated within an NC does not have a limit and increases with the increasing number of absorbed photons. However, experimental evidence does not confirm that simple model. For all the investigated Si NCs embedded in a SiO_2_ matrix (see the Materials and Methods section for more details on the investigated samples), we observed that under 405 nm (3.06 eV) cw excitation, the PL intensity, *I*_PL_, initially follows a linear dependence at low flux: *I*_PL_∝*φ*^*a*^ with *a*=1, where *φ* is the excitation photon flux and *a* is the growth rate. Above a certain threshold value for the excitation pump flux, the PL intensity growth rate decreases to a sublinear power dependence (*I*_PL_∝*φ*^*a*^ with 0<*a*<1; high flux). Complete saturation (*I*_PL_∝*φ*^*a*^ with *a*=0; high flux) is never achieved, even for the highest flux used in this study, for which multiple photons are absorbed per NC (see Figure 1a for an exemplary data set obtained under cw excitation). Moreover, the increase in PL above the expected saturation point is accompanied by a blueshift of the PL spectrum over (almost) the entire investigated flux range (Figure 1b).

Both the experimentally observed change in the PL intensity with the pump power and the accompanying blueshift of the PL spectrum are depicted in Figure 1 and clearly show two distinct regimes. This result violates the generally accepted model of excitonic PL in Si NCs, where efficient Auger interaction leads to the near-instant (picosecond time scale) quenching of multiple excitons localized in the same NC, so that a maximum of only a single electron-hole pair per NC can contribute to the PL^14^. Under assumptions of a fast Auger recombination, the dependence of the PL intensity, *I*_PL_, on the flux, *φ*, is^15^:


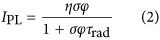


where σ is the optical absorption cross section at the pump wavelength, *τ*_rad_ is the time constant of radiative recombination and *η* is the internal quantum yield, which is defined as 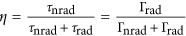, where *τ*_rad_ (Γ_rad_) and *τ*_nrad_ (Γ_nrad_) are the radiative and non-radiative recombination time constants (rates), respectively. Thus, at a high flux, the PL intensity should saturate at *η*/*τ*_rad_, which is clearly inconsistent with the experimental results.

In addition, the pump photon flux dependence of the PL peak position has two different ranges. The PL band blueshifts with the pump flux; however, the rate of this shift clearly decreases in the high-flux excitation range. The blue spectral shift at low powers is commonly attributed to the NC size dependence of the absorption cross section^14^, so the size distribution of excited NCs changes under different excitation fluxes. Large NCs have a larger absorption cross section^15^ and will therefore saturate at a lower excitation photon flux than the smaller NCs. This results in a blueshift of the PL spectrum. At even higher excitation photon fluxes, when all NCs are saturated regardless of their size, the PL spectrum will not shift any more due to the size-dependent absorption cross section. However, at high flux (with more than one exciton per NC), the experimental PL spectrum did not stabilize but continued to blueshift, albeit at a lower rate.

### Sample temperature

To explain the microscopic origin of the experimental findings of this study, we considered the effect of the high excitation photon flux on the sample temperature since the experimental observations indicate that at high flux, the sample temperature increases. At a high excitation photon flux, many photons are absorbed by a single NC, and most of the deposited energy is dissipated as heat, increasing the NC temperature. Under the highest flux used in this study, more than 100 photons were absorbed by a single NC within the exciton lifetime. Under the assumption of efficient Auger recombination and carrier-phonon scattering and thus the ‘effective presence’ of a single exciton per NC only, more than 300 eV of energy will be converted into heat in every NC within the effective exciton lifetime of approximately 100 μs. This readily explains the permanent damage of the investigated material and the initial irreversible decrease in the PL intensity, which can be inflicted by an extreme excitation flux (Supplementary Fig. S1). Of note, the necessary precautions were taken so that all of the results reported in this study were obtained under conditions in which no permanent change/damage to the samples occurred, and the results were fully reproducible on the same sample, which could be cycled multiple times. This is illustrated in Supplementary Fig. S1 in which the PL intensity of the sample was monitored upon prolonged and sequential exposure to the excitation laser beam. From these results, we conclude that no permanent change to the sample was inflicted under the conditions of the experiment, and in particular, its chemical composition remained constant, thus excluding these aspects as possible explanations of the results of the study. The situation is similar to that of freestanding Si particles whose temperature was shown to rise up to approximately 1350 K under intense cw laser illumination^16, 17, 18, 19, 20, 21, 22^. In addition, vibrational lifetimes can be significantly altered in nanostructures, leading to phonon localization and trapping, promoting an even further temperature increase^23^. The sample temperature can be conveniently estimated from Raman measurements^16, 17, 18, 19, 20^. However, in the present case, unlike in recalled studies, the relatively strong excitonic PL precludes direct observation, and an intensity comparison of the Stokes and anti-Stokes Raman modes, which is necessary for temperature evaluation.

**Figure 2:**
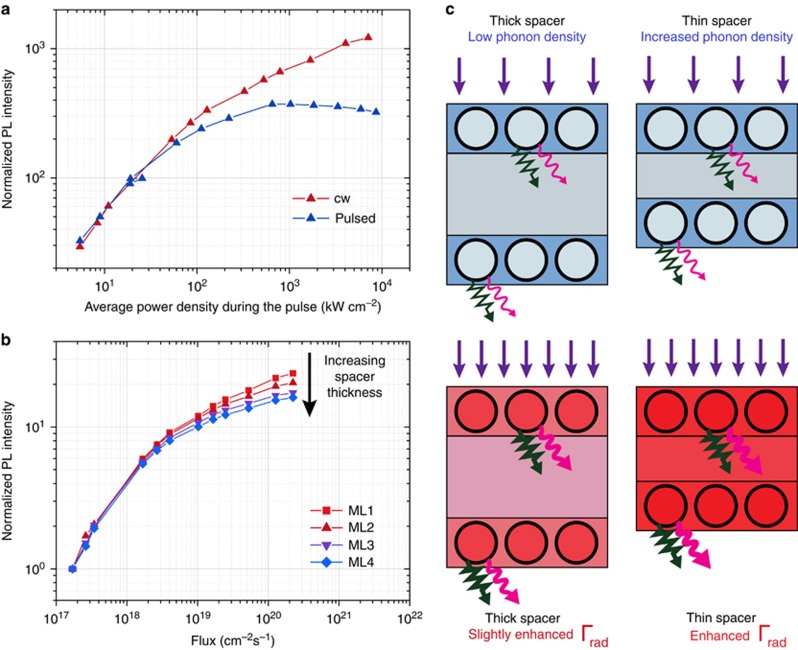
Evidence for laser-induced heating under continuous wave excitation. (**a**) Power dependence of the PL intensity (*λ*_det_=915 nm, *E*_det_=1.36 eV) of the ML sample (PECVD) with a spacer thickness of 1.6 nm (ML2) under 405 nm (3.06 eV) cw (red triangles pointing up) and 355 nm (3.49 eV) pulsed (blue triangles pointing up) excitation, depicted in double logarithmic representation. The results for the cw excitation are scaled to maximally overlay at low powers with the data obtained under pulsed excitation. (**b**) Normalized PL intensity (*λ*_det_=915 nm, *E*_det_=1.36 eV) versus excitation photon flux for the ML samples (PECVD) with spacer thicknesses of 1 (squares, ML1), 1.6 (triangles pointing up, ML2), 2.2 (triangles pointing down, ML3) and 2.8 nm (diamonds, ML4) under 405 nm cw excitation (scaled to maximally overlay the below-saturation points) in double logarithmic representation. (**c**) Schematic illustration (not to scale) of the discussed processes for samples with different spacer layer thicknesses (right and left column) and under low (top row) and high (bottom row) excitation photon fluxes. With increasing incident photon flux (purple arrows), the photon emission (magenta wavy arrows) and the phonon generation rate (dark green zigzag arrows) increases (indicated by the thickness of the symbols), whereas the phonon density increases for thinner spacers. Under low excitation photon flux, the phonon generation rate is low, and the spacer thickness-dependent phonon density does not lead to a strong enough temperature enhancement. Thus, the radiative emission rate, Γ_rad_, is not significantly influenced. Therefore, the below-saturation points in **b** are independent of the spacer thickness. The increase in the radiative rate becomes substantial at high temperature when the phonon density is high. Thus, at high flux, the phonon density, which is dependent on the spacer thickness, is an important parameter that governs the enhancement of the radiative emission rate. This can also be seen in **b**, where stronger saturation (lower radiative emission rate) occurs for thicker spacers (lower phonon density).

To verify sample heating as a possible origin of the additional PL, we compared the flux dependence of the PL intensity under 405 nm (3.06 eV) cw and 355 nm (3.49 eV) pulsed excitation. As shown in Figure 2a, in the latter case, complete saturation was observed. Because the Si NCs can rapidly dissipate energy between two consecutive excitation pulses, the photons are predominantly emitted not at elevated temperature. This result supports our notion that the additional growth of the PL under strong cw pumping could be related to the temperature increase of the sample. This was further confirmed by experiments on a series of structures featuring single Si NCs layers intercalated by spacers of pure SiO_2_ (Supplementary Fig. S2 for a schematic illustration). As shown in Figure 2b, the PL intensity saturation sets in as the spacer thickness increases. For ensembles of closely spaced Si NCs, one can expect that the thermal properties of the Si NC ensembles sensitively depend on the NC spacer thickness. For thinner spacers, more energy is absorbed and dissipated in the smaller volume of the ML structure, leading to stronger heating and a larger temperature increase (see Figure 2c for a schematic illustration). In Figure 2b, the normalized PL intensity is shown for a set of ML samples produced by PECVD with different spacer thicknesses but almost identical size distributions. For a smaller spacer thickness, there is a larger temperature increase and a more pronounced deviation from the expected saturation behavior (Figure 1). Thus, all these measurements are consistent with our hypothesis that sample heating is responsible for the additional PL.

The PL intensity can continue to grow above the expected saturation point, if either the radiative rate increases or the non-radiative rate decreases (Equation (2)). The latter typically increases with temperature due to the enhancement of multiphonon recombination for the larger phonon availability^24^. Therefore, the persistent increase in the PL intensity above the expected saturation point indicates an enhancement in the radiative rate.

### Effect of temperature on the radiative recombination rate

We now theoretically consider the effect of the temperature increase on the radiative excitonic emission in the Si NCs. The radiative recombination in bulk Si is assisted by phonons. The recombination rate in the bulk, Γ_rad_, can be presented as^25^:


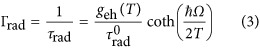


Here, 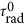 is the radiative recombination lifetime at zero temperature, neglecting the Coulomb interaction between electrons and holes, and *g*_eh_ is the effective Sommerfeld factor. The average optical phonon number is 
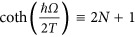
 with *N*=1/(*e*^*ħΩ*/*T*^−1), where *ħΩ*~60 meV~700 K is the characteristic optical phonon energy of Si, which accounts for the sum of the Stokes and anti-Stokes processes. It is close to unity at room temperature (*N*≪1) and increases at higher temperatures due to the growth of the average optical phonon number, resulting in (i) stimulation of the Stokes processes (~*N*+1) and (ii) the emergence of anti-Stokes processes with phonon absorption (~*N*). However, the total radiative rate in the bulk decreases with temperature because of the Sommerfeld factor, *g*_eh_(*T*), in Equation (3)^25, 26^.

The Sommerfeld factor describes the enhancement of the radiative rate due to the Coulomb attraction between electrons and holes, increasing their overlap matrix element. In the bulk, heating of electrons and holes leads to an increase in their kinetic energy and the effective electron-hole distance. Thus, the Coulomb enhancement of the radiative rate is suppressed, the Sommerfeld factor decreases, and the radiative rate in the bulk is quenched with temperature. In NCs, the situation is qualitatively different. The carriers are confined and cannot spread in real space when being heated. The quantitative measure of the confinement strength is the ratio between the NC size and the exciton diameter. For all of the Si NCs considered in this study, the diameters are smaller than twice the bulk exciton radius, 2*a*_B_≈9 nm^27^. Hence, the Coulomb interaction energy becomes weaker than the quantum confinement energy, and the Sommerfeld factor can be neglected for NCs. The temperature dependence of the radiative rate in the NCs differs strongly from the bulk, increasing with temperature. Namely, for the considered sizes of the NCs, the recombination remains phonon-assisted^28^. The radiative rate is then solely determined by the phonon population, 
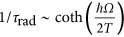
, and it should increase with temperature due to the Bose stimulation of phonon emission. The increase in the radiative rate becomes substantial for temperatures that are comparable to the optical phonon energy, *ħΩ*~700 K.

**Figure 3:**
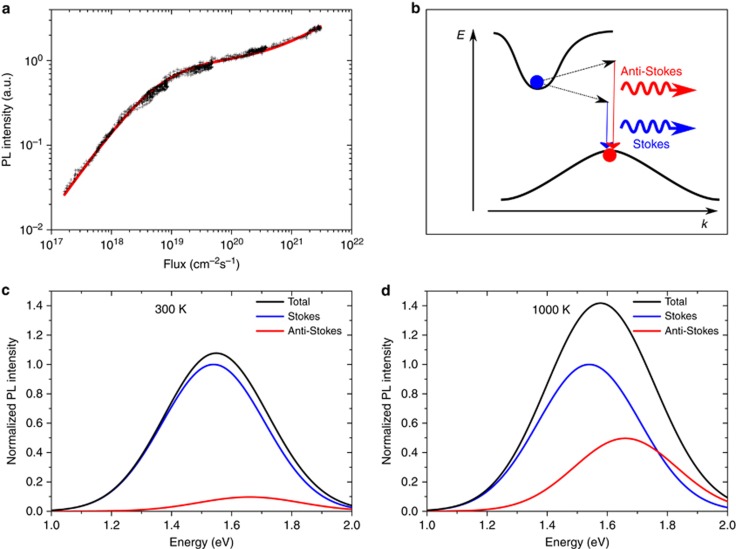
Stokes and anti-Stokes emission. (**a**) The PL intensity (of a co-sputtered sample, *λ*_exc_=870 nm, *E*_det_=1.43 eV) for Si NCs in a SiO_2_ sample under 405 nm (3.06 eV) cw excitation with a fit to the proposed theoretical model, taking into account laser-induced heating of the Si NCs and resulting in an enhancement of the radiative recombination rate at high excitation photon flux. (**b**) Schematic illustration of radiative recombination with the absorption (anti-Stokes) or emission (Stokes) of a phonon to compensate for the momentum mismatch. The Stokes and anti-Stokes emission are separated from each other by two times the optical phonon energy (120 meV). (**c**) Schematic illustration of the PL spectrum (black), normalized to the peak intensity of the Stokes spectrum for a temperature of 300 K with contributions from the Stokes (blue) and anti-Stokes (red) processes. (**d**) Schematic illustration of the PL spectrum (black) normalized to the peak intensity of the Stokes spectrum for a temperature of 1000 K with contributions from the Stokes (blue) and anti-Stokes (red) processes. The relative contribution of the anti-Stokes emission to the total PL spectrum increases when the temperature rises, leading to an effective blueshift of the PL spectrum at a high photon excitation flux.

To estimate whether such a strong heating of the NC layers is plausible, we considered the balance between the absorption of the laser power and the heat escape through the layer boundary. Our analysis of the heat equation^29^ in the film geometry is described in additional detail in the Supplementary Information. The temperature of the sputtered layer with a thickness of *L*~1 μm has been evaluated as *T*~300 K+*P*_abs_*ξ*/(2*πkL*), where *P*_abs_ is the absorbed laser power, *k* is the heat conductivity and 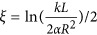 is the dimensionless factor of the order of unity, depending on the excitation spot radius, *R*, and the heat transfer through the film boundary, *α*. In crystalline Si and SiO_2_ the heat conductivity coefficients are equal to *k*~140 W/(m·K) and *k*~1 W/(m·K), respectively. This value leads to a relatively weak temperature increase of the order of approximately 100 K at the highest investigated flux under 405 nm cw excitation. However, it has been shown in Refs. 30, 31 and others that the heat conductivity becomes more than 10 times lower in thin SiO_2_ films with a thickness on the order of microns compared to bulk SiO_2_. In the samples investigated in this study, the effective heat conductivity should be mostly determined by SiO_2_ since its volume fraction in the studied samples exceeds 75 %. Thus, we can reasonably assume a fivefold suppression of the heat conductivity in the considered sputtered ML structures, where each active and passive layer has a thickness below 10 nm, *k*~0.3 W/(m·K). As a result, we arrive to a temperature of ~1000 K and a radiative enhancement rate of approximately three times at the highest investigated photon flux, which is in quantitative agreement with our experimental findings. In Figure 3a, the experimental data of the co-sputtered sample are shown with the theoretical model derived from Supplementary Equations S10 and S11, Equations (2) and (3).

For a given heat conductivity, the temperature increase reduces with the total thickness of the sputtered ML film and is therefore suppressed for thicker spacers between NC layers, which is also in good agreement with the experimental results in Figure 2b. Of note, in the proposed treatment, we only take into account carrier interactions with optical phonons. Possible contributions from acoustic phonons with lower characteristic energies will lead to a rate enhancement even at lower temperatures. Thus, our results should be considered as an estimation of the lowest possible radiative rate enhancement.

The laser-induced heating of the sample and the related presence of phonons also have a pronounced effect on the PL spectrum. At low temperature, the exciton recombination predominantly involves the release of phonons, resulting in a spectrally redshifted PL spectrum compared to the exciton energy (Stokes replica). At higher temperatures, radiative recombination accompanied by phonon absorption (anti-Stokes emission) also appears, resulting in an effective blueshift of the PL spectrum at a high excitation photon flux (see Figure 3 for schematic illustrations). This effect was indeed experimentally observed—see the persistent blueshift of the PL spectrum above the expected saturation point in Figure 1b. The appearance of the blueshift for the high-flux regime cannot be explained by the NC size-dependent absorption cross section as discussed earlier and provides the most direct support for the proposed interpretation of the additional emission in terms of its thermal stimulation.

### Alternative mechanisms

For completeness, we will now discuss alternatives for the possible origin of the continuation of the PL intensity increase and the blueshift of the PL spectrum above the saturation point. One possibility for the additional PL at high pumping flux is the excited-state emission, as proposed by G Faraci *et al.* to explain the giant superlinear PL intensity in crystalline Si grains that were approximately 100 nm in size^17^. The necessary condition in that case is that the radiative rate is significantly larger and/or the Auger recombination rate reduces for hot compared to ground-state excitons. However, it is well established that the phonon-assisted radiative recombination rates are nearly identical for low-lying excited states since the matrix elements of the interaction between carriers and optical phonons are not strongly modified at low excitation energies.

**Figure 4:**
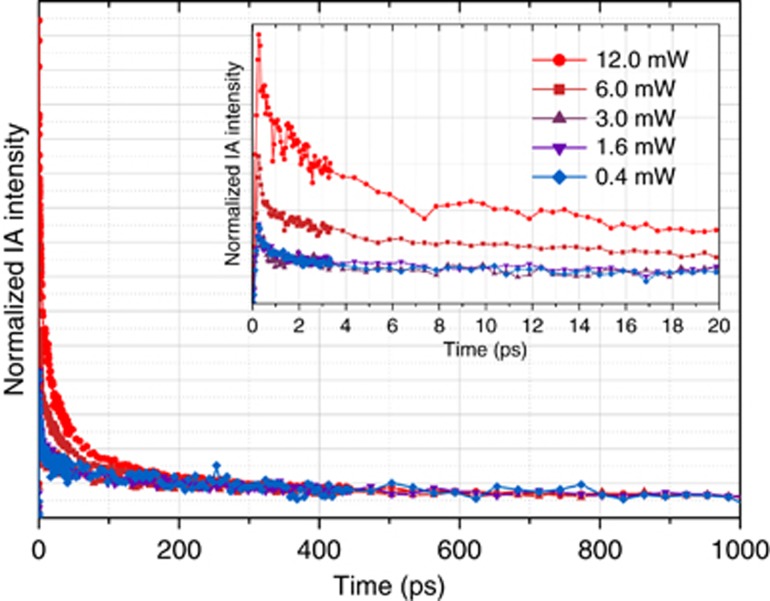
Transient-induced absorption dynamics for several excitation photon fluences. Normalized transient IA dynamics of the same sample as depicted in Figure 1 under 500 nm (2.48 eV) excitation measured at probe wavelengths near 1250 nm (0.99 eV, obtained by integrating the signal from 1200 to 1300 nm) for pump pulse fluences of 0.4 (diamonds), 1.6 (triangles pointing down), 3.0 (triangles pointing up), 6.0 (squares) and 12.0 mW (circles), depicted in linear representation. Transients are normalized to an equal number of absorbed photons. In the inset, the normalized IA transients zoomed into the first 20 ps are shown.

Another way to generate additional photons above the saturation level predicted by the commonly accepted model is to allow radiative recombination from two (biexciton) or more electron-hole pairs in a single NC, as proposed by D Kovalev *et al.*^15^, F Pevere *et al.*^32^ and others. Our additional analysis indicates, however, that the biexciton model cannot explain the observed PL enhancement. Applying the biexciton model to our data yields values of 100–400 ns for the non-radiative lifetime of the biexciton. This is controlled by the Auger process, as previously mentioned (see Supplementary Information for further details and a corresponding fit to our data). Although Auger lifetimes on the order of a nanosecond have been occasionally proposed by theory in the past^13, 33^ and more recently for NCs with certain ‘resonance’ dimensions^34^, they are much different from those obtained in more recent calculations^35^ and, most importantly, are inconsistent with multiple experimental data^36, 37, 38, 39, 40^. To establish the Auger recombination time in our sample, we carefully measured it using a femtosecond pump-probe technique. The NCs were excited by 500 nm (2.48 eV) photons, and a near-infrared continuum (850–1600 nm, 0.78–1.46 eV) was used as the probe pulse. Following the procedure introduced by Klimov *et al.*^41^, we cautiously measured the transient IA dynamics for several excitation intensities of the pump, as shown in Figure 4. From this data, we conclude that the biexciton lifetime for our ML sample is shorter than 100 ps, which is in agreement with previous reports^36, 37, 38, 39, 40^. This value is much smaller than the Auger lifetime necessary to explain the experimentally observed PL enhancement within the framework of the biexciton model. Therefore, we discount the idea that biexciton emission is responsible for the PL enhancement above the expected saturation point observed in this study.

## Conclusions

In conclusion, we demonstrated that the radiative rate of exciton recombination in Si NCs can be effectively enhanced by an increase in temperature. In particular, a threefold enhancement was demonstrated upon laser-induced heating in ML structures of Si NCs embedded in SiO_2_. Future research will need to test the limits of this effect and determine whether purposeful phonon management in Si nanostructures can yield radiative recombination rates with application potential. For example, the insulation of the NCs or the use of a matrix with a better or worse thermal conductivity can affect the radiative lifetime and lower the onset of the radiative rate enhancement. Moreover, the possibility of phonon injection should be investigated to increase the radiative rate without inducing damage to the sample due to heat generation. The present study identifies an important new advantage of Si NCs and shows that dedicated phonon management on the nanoscale could offer a possible avenue toward enhancement of the optical faculty of Si for the much desired Si photonics. Moreover, since the photon fluxes under which the observed increase of the radiative emission rate occurs are comparable to those in concentrator photovoltaics, the current findings could also be relevant for the use of Si NCs in future generations of solar cells.

## Author contributions

EdJ, HR, JV, and TG conceived the project and designed the experiments; EdJ, HR and JV performed the PL experiments and contributed to data analysis; MT performed the IA experiments; AP and IY provided theoretical interpretations and modeling; AC contributed to the finalization of the manuscript; EdJ and TG co-wrote the manuscript; TG supervised the project; All authors discussed the results and commented on the manuscript.
