# Supplementary material for: Thermally stimulated exciton emission in Si nanocrystals
Source: Light Sci Appl. 2018 Jan 26;7:17133–. doi: 10.1038/lsa.2017.133 (PMC6107050; doi:10.1038/lsa.2017.133)
Supplement: Supplementary Information [file lsa2017133x1.docx]

**Supplementary Information**

**“Thermally stimulated exciton emission in Si nanocrystals”**

Elinore M. L. D. de Jong, Huub Rutjes, Jan Valenta, M. Tuan Trinh, Alexander N. Poddubny,

Irina N. Yassievich, Antonio Capretti, and Tom Gregorkiewicz

Photoluminescence intensity as function of illumination time


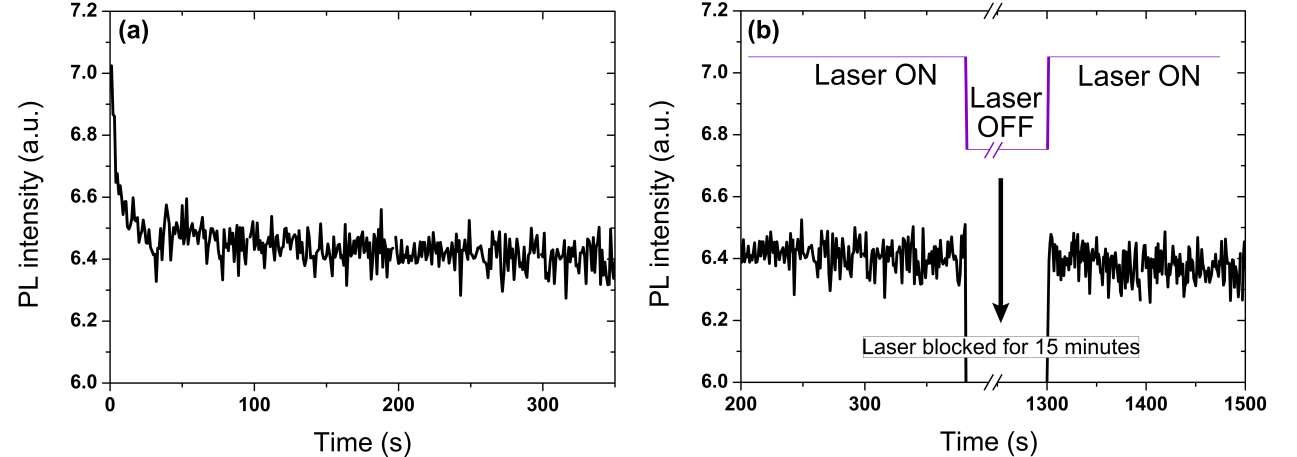


**Figure SI1. Photoluminescence intensity as function of illumination time.** (a) When the sample is exposed to the highest excitation photon flux (*λ*_exc_ = 405 nm, *E*_exc_ = 3.06 eV) used in this study, the PL intensity decreases towards a constant value within 100 s. (b) This value of the PL intensity remains constant and is reproduced after the laser is blocked for 15 min, indicating that the decrease of the PL intensity is permanent, so irreversible. In order to exclude the irreversible behavior depicted in panel (a) from our results, in this study we have always laser-illuminated the sample for several minutes before each measurement, until the PL intensity stabilized, and during the measurement itself we have checked the reproducibility of the data by ramping in laser power back and forth. All the results in this paper are obtained under conditions where no permanent change to the samples occurs and are reproducible on the same sample. Hence the irreversible initial decrease of the PL intensity depicted in panel (a) is excluded by the experimental procedure and cannot in any way be responsible for the observed experimental results.

Theoretical evaluation of the temperature of the multilayer film

Our goal is to estimate the temperature increase, *T’*, of the ML film with Si NCs, shown

in Figure SI2, due to the cw laser illumination. To this end, we solve the heat transport

equation^1^:

|  | $div k\left( z \right)grad T^{'}\left( \rho,z \right)=-Q\left( \rho,z \right) \mathrm{with} k\left( z \right)=\left\{ \begin{aligned} 0, z<0 \\ k, 0<z<L \\ 0, \mathrm{otherwise} \end{aligned} \right\}$, | (SI1) |
| --- | --- | --- |

with boundary condition

|  | $-k\frac{dT^{'}}{dz}=\alpha T^{'}$, | (SI2) |
| --- | --- | --- |

and both at the upper and lower boundaries of the film $z=0$ and $z=L.$ Here, *α* is the heat transfer coefficient through the boundary. We assume a homogenous heat source with radius *R*,

|  | $Q\left( \rho,z \right)=\left\{ \begin{aligned} Q 0<z<L, \rho<R \\ 0, \mathrm{otherwise} \end{aligned} \right\}$ | (SI3) |
| --- | --- | --- |

with the heat volume density $Q=\frac{P}{\pi R^{2}L}$ and *P* being the absorbed power. The homogenous approximation is valid, since the film thickness is smaller than the light absorption length. This is verified by the measured absorption, that is equal to ~16 %.

The system of Eq. SI1 to SI3 can be solved using the Green function of the heat equation,

|  | $div k\left( z \right)grad G\left( \boldsymbol{\rho}-\boldsymbol{\rho}^{\boldsymbol{'}},z,z^{'} \right)=\delta(\boldsymbol{r}-\boldsymbol{r}^{\boldsymbol{'}})$, | (SI4) |
| --- | --- | --- |

that is found by means of the Fourier expansion along the coordinates $x$ and $y$:

|  | $G=\iint\frac{d^{2}\boldsymbol{q}}{\left( 2\pi\right)^{2}}\frac{1}{2\boldsymbol{q}}e^{i\boldsymbol{q}\left( \boldsymbol{\rho}-\boldsymbol{\rho}^{'} \right)}g_{q}\left( z \right), \mathrm{with} g_{q}\left( z \right)=e^{-q\left\vert z-z^{'} \right\vert}+2A\cosh q\left( z-\frac{L}{2} \right) \mathrm{and}0<z<L$ | (SI5) |
| --- | --- | --- |

The coefficient $A$ is determined from the boundary conditions as:

|  | $A=\frac{(kq-\alpha)e^{-q(L/2-z^{'})}}{2[\alpha cosh( qL/2)+kqsinh( qL/2)}$. | (SI6) |
| --- | --- | --- |

The temperature increase reads:

|  | $T^{'}\left( \boldsymbol{r} \right)=\int d^{3}r^{'}G\left( \boldsymbol{\rho}-\boldsymbol{\rho}^{\boldsymbol{'}},z,z^{'} \right)Q(\boldsymbol{\rho},z^{'}).$ | (SI7) |
| --- | --- | --- |

Substituting the Green function, we find the temperature increase in the center of the spot as:

|  | $T^{'}\left( z,\rho=0 \right)=\frac{QR}{k}\int_{0}^{\infty} dq\frac{J_{1}\left( qR \right)}{q^{2}}F\left( q \right),$  with $F\left( q \right)=1-\frac{\alpha\cosh q(z-L/2)}{\alpha\cosh(qL/2)+kq\sinh(qL/2)}$ | (SI8) |
| --- | --- | --- |

Next, we present an approximate analytical estimation of the integral taking into account that the pump spot radius $R=10 \mu m$ is much larger than the film thickness $L \sim0.85 \mu m$. The expression under the integral in Eq. SI8 has two factors, namely the form factor containing the Bessel function and the factor $F(q)$ depending on the heat transfer coefficient. We expand the first factor in the series over the small parameter $qR$ and assume that the heat conductivity is weak, $F(q)\approx1.$ This yields the following integral for the temperature increase:

|  | $T^{'}\left( \boldsymbol{r} \right)=\frac{QR^{2}}{2k}\int\frac{dq}{q},$ | (SI9) |
| --- | --- | --- |

that diverges at both lower and upper limits. The integral can be made convergent by introducing the cutoffs at $q=0$ and $q=\infty$by comparing it with the exact integral (Eq. SI8), that is regular both at $q=0$ and $q=\infty$. The upper cutoff in Eq. SI9 is at $q_{\max}\sim1/R$, where the Bessel function starts decaying. The lower cutoff in Eq. SI9 is at the wave vector $q=q_{\min}=\sqrt{2\alpha/kL}$, where the factor $F(q)$ becomes small. As a result, the temperature increase can be estimated as:

|  | $T^{'}\left( \boldsymbol{r} \right)=\frac{QR^{2}}{2k}\xi\equiv\frac{P\xi}{2\pi kL},$ | (SI10) |
| --- | --- | --- |

with the dimensionless coefficient:

|  | $\xi=\ln\frac{q_{\max}}{q_{\min}}=\frac{1}{2}\ln\left( \frac{kL}{2\alpha R^{2}} \right).$ | (SI11) |
| --- | --- | --- |

The exact value of the heat transfer coefficient at the film boundaries is not known, since it

depends on the microscopic structure of the sputtered MLs. We perform the numerical estimation using the values typical for the radiative transfer, $\alpha\sim{\sigma T}^{3}$, where $\sigma=5.7\times{10}^{-8} W/(m^{2}{\cdot K}^{4})$ is the Stefan-Boltzmann constant. For $T=1000 K,$ we obtain $\alpha\sim60 W/{(m}^{2}\cdot K)$. Using the value $k \sim0.3 W/(m\cdot K)$, we arrive at $T \sim1000 K$ in Eq. SI10, in agreement with the numerical evaluation of the integral (Eq. SI8). The calculated temperature of the ML film with the Si NCs (co-sputtered, depicted in Figure 1) as function of the excitation photon flux is shown in Figure SI3, in double logarithmic representation.


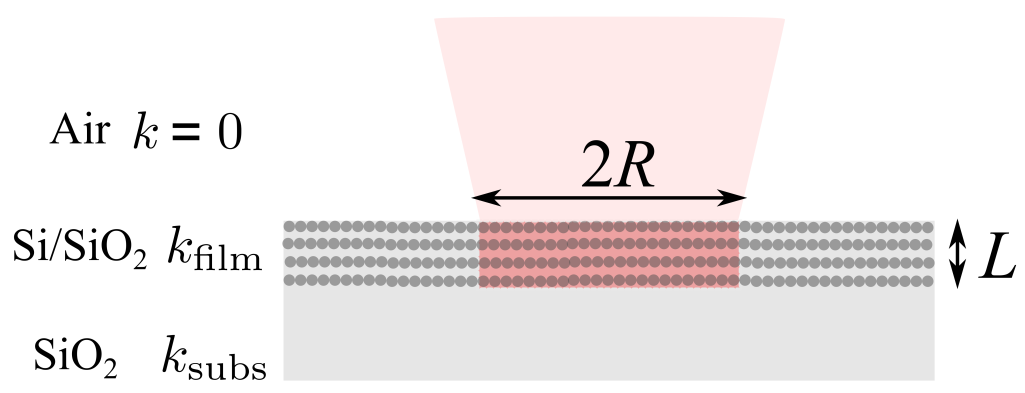


**Figure SI2. Sketch of the film with Si nanocrystals under the focused laser illumination.** The optically active layer, on top of a SiO_2_ substrate with heat conductivity *k*_subs_, has a ML structure with thickness *L* and a heat conductivity *k*_film_. It is exposed to a laser beam with a pump spot radius R.


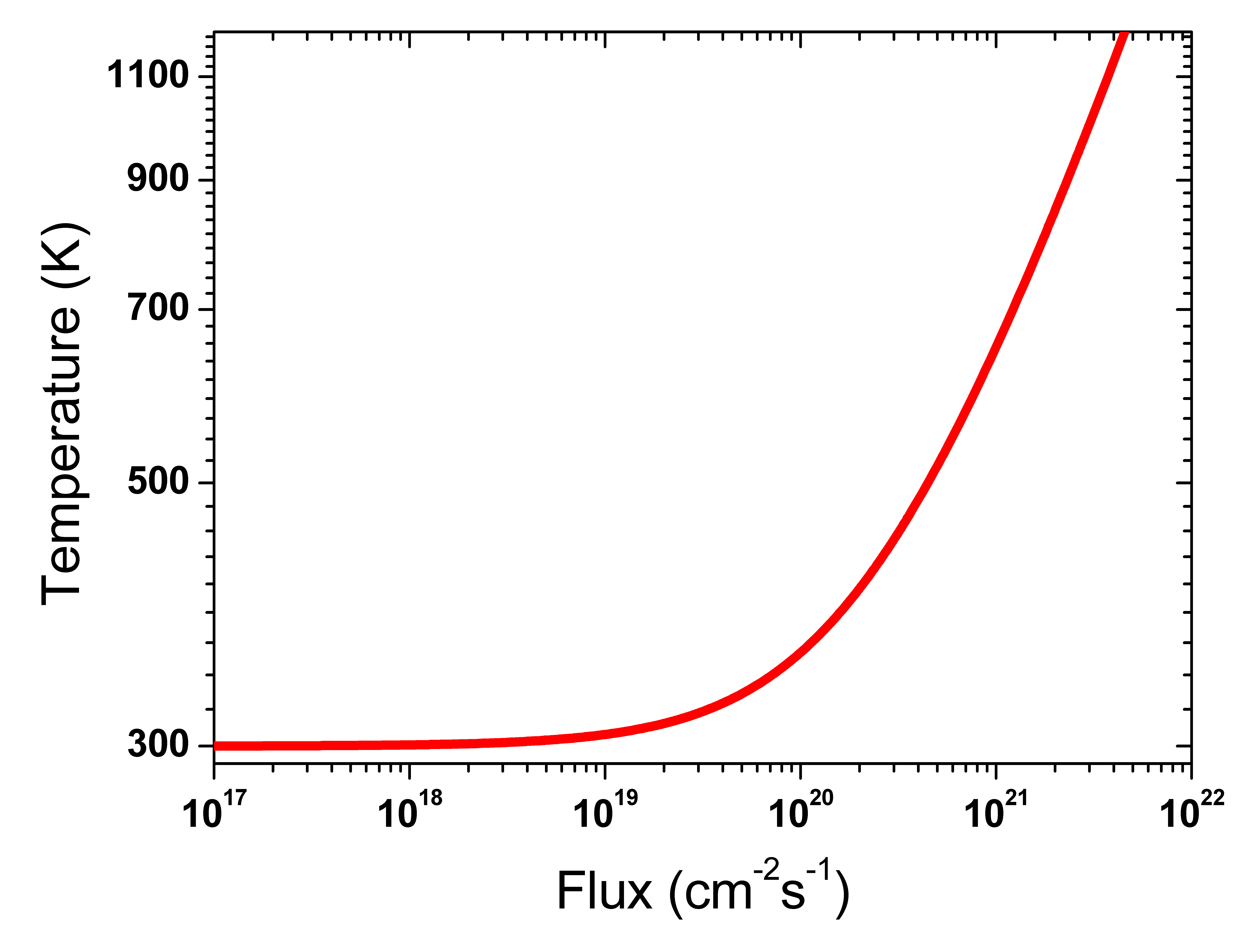


**Figure SI3. Theoretical evaluation of the temperature.** The calculated temperature of the ML film with Si NCs as function of the excitation photon flux, in double logarithmic representation.

Determination of the biexciton lifetime

In general, a NC can be either in a ground or excited state containing one, two or more excited electron-hole pairs (excitons). Let us consider a universal model with ($n+1$)-states (where $n$ is the maximum number of excitons in a NC). An electron-hole pair is created by the absorption of a photon and recombines with a characteristic lifetime, either radiatively or non-radiatively. A schematic diagram of this in the case of a three-state system is given in the inset of Figure SI4. The rate equation for the probability $P_{i}$ to have *i* ≥ 0 excitons is of the form:

|  | $\frac{dP_{i}}{dt}=\sigma\varphi\left( P_{i-1}-P_{i} \right)+\frac{P_{i+1}}{\tau_{i+1}}-\frac{P_{i}}{\tau_{i}}$*,* | (SI12) |
| --- | --- | --- |

where $\sigma$ is the absorption cross-section, $\varphi$ the excitation photon flux, $\tau_{i}$ is the effective lifetime of the i^th^ exciton, $P_{-1}=0$ and $P_{tot}=\sum P_{i}=1$. One approach to solving this in the steady-state case is the following. In steady state we have $\frac{dP_{i}}{dt}=0$ and we can write Eq. SI12 as the homogenous system:

|  | $\mathbf{0}=M\mathbf{P}$, | (SI13) |
| --- | --- | --- |

where $\mathbf{P}=\{P_{0},$*…,*$P_{n}\}$ and M are $n \times n$ matrices containing $\pm\sigma\varphi$ and $\pm\frac{1}{\tau_{i}}$ at the appropriate positions. The general solution for an $n$-state system is:

|  | $\left\{ \begin{aligned} P_{0}=1/\Delta i=0 \\ P_{i}=\Pi_{k=1}^{i}\sigma\tau_{k}\varphi/\Delta1\leq i\leq n \end{aligned} \right\}$ | (SI14) |
| --- | --- | --- |

with $\Delta=1+{\sum_{i=1}^{n} (\Pi}_{k=1}^{i}\sigma\tau_{k}\varphi)$. The effective lifetime is:

|  | $\frac{1}{\tau_{i}}= \frac{1}{\tau_{i,\mathrm{rad}}}+ \frac{1}{\tau_{i,\mathrm{nrad}}}$, | (SI15) |
| --- | --- | --- |

where $\tau_{i,\mathrm{rad}}$ and $\tau_{i,\mathrm{nrad}}$ are the radiative and non-radiative lifetime, respectively. The PL (average number of total emitted photons per second) from an $n$-state system is given by:

|  | $I_{\mathrm{PL}}=N^{*}\sum_{i=1}^{n} \frac{P_{i}}{\tau_{i,\mathrm{rad}}}$, | (SI16) |
| --- | --- | --- |

where $N^{*}$ is the number of emitting NCs. It is also convenient to define:

|  | ${\varphi_{i}\equiv(\sigma\tau_{i})}^{-1}.$ | (SI17) |
| --- | --- | --- |

When we do not allow radiative recombination of higher states (more than one electron-hole pair in a single NC), the PL under cw excitation is given by:

|  | $I_{PL,2}=A_{2}\frac{\varphi}{1+\varphi/\varphi_{1}}$, | (SI18) |
| --- | --- | --- |

where $A_{2}$ is a proportionally factor. This is the generally accepted model, where the PL intensity saturates completely as only a single exciton state gives photon emission. Since we do not observe such a complete saturation, it is evident that the observed excitation photon flux dependence of the PL intensity cannot by fitted by this equation.

Allowing for radiative recombination of biexcitons (i.e. two electron-hole pairs in a single NC), where there are four pathways for an electron and hole of the two electron-hole pairs to recombine and we therefore assume $\tau_{2,\mathrm{rad}}=\frac{\tau_{1,\mathrm{rad}}}{4}$ , leads us to the following formula for the PL intensity:

|  | $I_{PL,3}=A_{3}\frac{\varphi+4\varphi^{2}/\varphi_{2}}{1+\varphi/\varphi_{1}+\varphi^{2}/{{(\varphi}_{1}\varphi}_{2})}$, | (SI19) |
| --- | --- | --- |

where $A_{3}$ is a proportionality constant. This formula allows us to fit to power-dependent PL intensity data (see Figure SI4). From the fit we can determine the recombination time of biexcitons. Using the value of *ρ* = $\varphi_{2}$/$\varphi_{1}$ = $\tau_{1}$/$\tau_{2}$ ~490 for the sample depicted in Figure 1 together with the effective lifetime of the exciton ($\tau_{1}$~130 μs), which we have determined from the experimentally measured PL decay dynamics, we arrive to a value of approximately 265 ns for the effective PL lifetime of the biexciton. The radiative biexciton lifetime is in the microseconds range, since $\tau_{2,rad}=\frac{\tau_{1,\mathrm{rad}}}{4}=\frac{\tau_{1}}{4\eta_{1}}$ where $\eta_{1}$ is the quantum efficiency of the exciton, which has a value between 0 and 1. Using this information together with Eq. SI15, we can approximate *τ*_2_ ~*τ*_2,nrad_. Therefore, we can assume that the method probes the non-radiative lifetime of the biexciton, which is usually directly linked to the Auger recombination lifetime. This leads to a value of approximately 265 ns for the Auger lifetime of the sample depicted in Figure 1. Similar values are found for other samples. Thus, applying the biexciton model to our data, we arrive to values of more than 100 nanoseconds for the non-radiative lifetime of the biexciton.


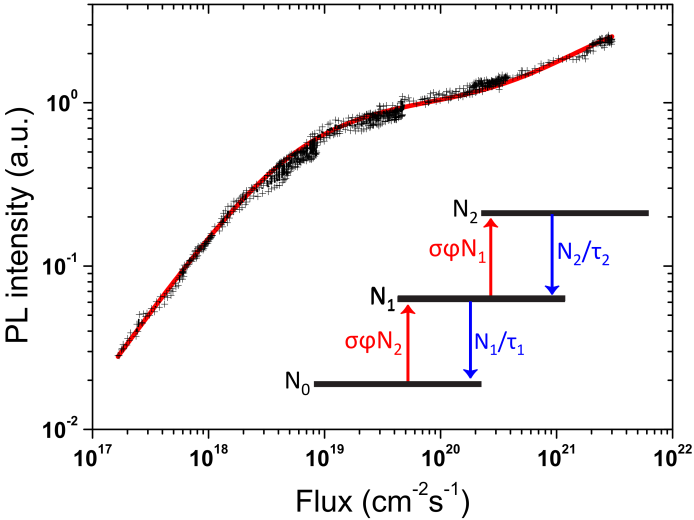


**Figure SI4. Typical flux dependence of the photoluminescence intensity for a Si nanocrystals in SiO_2_ sample under 405 nm (3.06 eV) continuous wave excitation with a fit to the biexciton model.** The PL intensity (*λ*_det_ = 870 nm, *E*_det_ = 1.43 eV) over more than four orders of magnitude of the excitation pump flux in double logarithmic representation with a fit to Eq. SI19 (red curve) of the same sample as depicted in Figure 1. A schematic diagram of three-state system is shown in the inset, as described by Eq. SI12.

Additional experimental data on the flux dependence of the photoluminescence


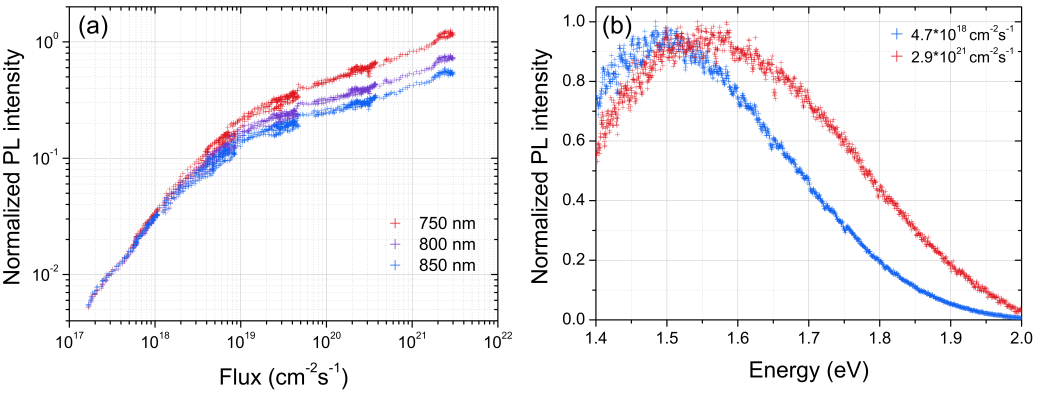


**Figure SI5. Flux dependence of the photoluminescence intensity for a Si nanocrystals in SiO_2_ sample under 405 nm (3.06 eV) continuous wave excitation.** (a) The PL intensity of the same sample as depicted in Figure 1 at *λ*_det_ = 750 (*E*_det_ = 1.65 eV, red), *λ*_det_ = 800 (*E*_det_ = 1.55 eV, violet) and *λ*_det_ = 850 nm (*E*_det_ = 1.46 eV, blue) as function of the excitation photon flux, depicted in a double logarithmic representation. The curves have been scaled vertically to overlap at the low flux. (b) Two PL spectra of the sample depicted in Figure 1 at relatively low (blue) and high (red) excitation fluxes.

*
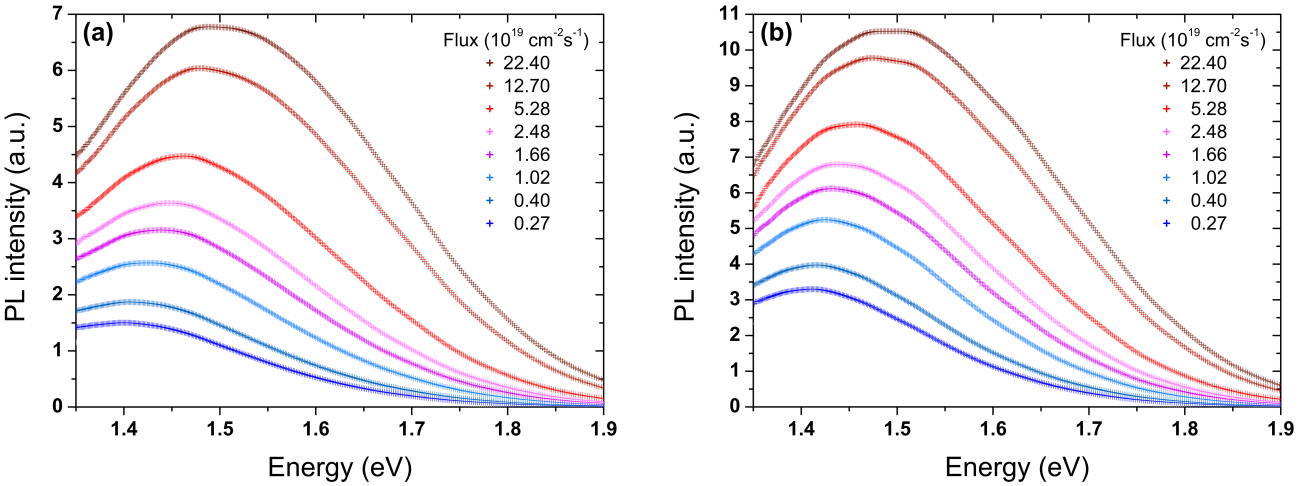
*

**Figure SI6. Exemplary photoluminescence spectra for several excitation photon fluxes under 405 nm (3.06 eV) continuous wave excitation.** (a) The PL spectra for eight excitation fluxes of the ML PECVD sample ML1 with a spacer thickness of 1 nm. (b) Flux dependence of the PL spectra for the ML PECVD sample ML4 with a spacer thickness of 2.8 nm.

**REFERENCES**

1. Lienhard IV JH, Lienhard V JH. *A Heat Transfer Textbook.* Phlogiston Press: Cambridge, MA, 2008.
